# Membrane-Assisted Electrochemical Removal of Mg^2+^ and Ca^2+^ from Lithium Brines: Effects of Temperature and Current Density Through a Zeta Potential Approach

**DOI:** 10.3390/membranes15090250

**Published:** 2025-08-25

**Authors:** Alonso González, Geovanna Choque, Mario Grágeda, Svetlana Ushak

**Affiliations:** 1 Center for Advanced Study of Lithium and Industrial Minerals (CELiMIN), Universidad de Antofagasta, Campus Coloso, Av. Universidad de Antofagasta, Antofagasta 02800, Chile; alonso.gonzalez@uantof.cl (A.G.); geovanna.choque.guisbert@ua.cl (G.C.); svetlana.ushak@uantof.cl (S.U.); 2 Departamento de Ingeniería Química y Procesos de Minerales, Universidad de Antofagasta, Av. Universidad de Antofagasta, Antofagasta 02800, Chile

**Keywords:** magnesium removal, lithium recovery, electrodialysis, zeta potential, Mg(OH)_2_, Ca(OH)_2_, energy consumption

## Abstract

Understanding surface charge behavior is essential for improving ion separation during lithium brine treatment. This paper investigates the performance of a three-compartment electrodialysis system designed for the selective removal of divalent cations (Mg^2+^ and Ca^2+^). The relationship between zeta potential and the recovery of Li^+^, Na^+^, and K^+^ is analyzed. Zeta potential measurements at various pH values showed that Mg(OH)_2_ particles maintained a positive charge. The system facilitated the precipitation of Mg(OH)_2_ and Ca(OH)_2_ via electrochemically generated OH^−^ ions. The specific electrical energy consumption was evaluated for each operating condition. The results showed that the zeta potential of the precipitates was affected by both the current density and temperature. This influenced lithium losses due to brine entrapment within the precipitated solids. At 600 A/m^2^ and 50 °C, more than 99% of Mg^2+^ and Ca^2+^ were removed, and more than 90% of lithium was recovered, with a specific electric energy consumption of 2.58 kWh per kilogram of Li recovered. The system also generates HCl as a valuable by-product, which improves the sustainability of the process. This study provides a new framework for improving the energy efficiency of lithium purification from brines and lithium recovery.

## 1. Introduction

The growing demand for lithium for the production of lithium-ion batteries has driven the development of more efficient and sustainable technologies for its extraction from natural brines and the production of lithium compounds. Among these compounds, LiOH is preferred for use in high-performance cells due to its greater thermal stability and better electrochemical performance [1]. Nevertheless, the direct production of LiOH from lithium chloride brines using electromembrane processes presents technical challenges [2,3], particularly due to the presence of divalent impurities such as magnesium (Mg^2+^) and calcium (Ca^2+^). These cations tend to precipitate as Mg(OH)_2_ and Ca(OH)_2_ and may foul ion exchange membranes, decreasing both system efficiency and membrane lifespan [4,5]. This problem is also observed in brines treated using direct lithium extraction (DLE) processes, which may co-extract unwanted monovalent and divalent cations, requiring additional purification steps [6,7].

Therefore, the development of purification technologies capable of selectively removing Mg^2+^ and Ca^2+^ from brines without compromising lithium recovery is essential for the sustainable production of LiOH through electrochemical methods [8,9]. Recent studies have emphasized the potential of integrating membrane-assisted electrochemical systems for resource recovery and desalination, highlighting their role in sustainable brine management [10,11].

In the traditional method of purifying lithium brine, Mg^2+^ and Ca^2+^ are removed as hydroxides and carbonates using lime slurry (Ca(OH)_2_) and soda ash (Na_2_CO_3_). While this method is effective, it has the disadvantage of diluting the brine and reducing lithium concentration in addition to adding Na^+^ as an impurity [12]. Alternative techniques for the removal of Mg^2+^ and Ca^2+^ from brine include nanofiltration membranes [13], ion exchange resins [14], and monovalent-selective membranes [15], all of which can be integrated into direct lithium extraction processes [16,17].

In this study, the removal method of interest is the electrochemical precipitation of Mg^2+^ and Ca^2+^ through electrochemical alkalinization. This approach utilizes the water reduction half-reaction at the cathode to generate hydroxide ions (OH^−^), which induce the precipitation of Mg(OH)_2_ and Ca(OH)_2_ [2,17]. The generation of OH^−^ ions acts similarly to the addition of a base. A low current density mimics the gradual addition of a weak base, while a high current density has an effect analogous to adding a strong base such as NaOH.

This method enables alkalinization of the brine and selective removal of Mg^2+^ and Ca^2+^ without the need for chemical additives, thereby avoiding brine dilution and the addition of impurities such as Na^+^ or Ca^2+^. Ion exchange membranes are used to facilitate the transport of anions, mainly Cl^−^, which are replaced by OH^−^ to maintain electroneutrality. Additionally, this method enables the recovery of by-products such as HCl or NaCl [2].

In previous studies, Nieto et al. [18] investigated a three-compartment electrodialysis system for the removal of Mg^2+^ from lithium-containing brines. In this system, the brine was used as the catholyte, focusing on the magnesium removal while simultaneously recovering Mg(OH)_2_ solids as a value-added product. These initial experiments demonstrated the feasibility of the technology as a proof of concept, successfully removing Mg^2+^ from three different brine compositions. One major challenge identified was the tendency of Mg(OH)_2_ precipitates to trap brine volumes, leading to contamination of the recovered solid.

In a more recent study [2], a similar three-compartment system was adapted to treat complex lithium-rich solutions obtained from direct lithium extraction (DLE). The main objective was to remove Mg^2+^ and Ca^2+^ by precipitating them as Mg(OH)_2_ and Ca(OH)_2_ while minimizing lithium losses. The experiments explored the effects of different brine concentrations, Na/Li molar ratios, and volumetric flow rates on Mg^2+^ and Ca^2+^ removal and lithium recovery. The study aimed to obtain a lithium concentration of 1% by weight, suitable for subsequent conversion to Li_2_CO_3_ or LiOH.

The most favorable results, in terms of effective Mg^2+^ and Ca^2+^ removal and minimal lithium loss, were achieved using concentrated brines with high Na/Li ratios and applying a current density of 1200 A/m^2^. Under these conditions, the specific electrical energy consumption (SEC) ranged from 27 to 34 kWh per kilogram of lithium recovered. These results suggest that, although high current densities enhance divalent cation removal, they lead to a substantial increase in energy demand, indicating the need for process optimization to improve energy efficiency while maintaining lithium selectivity.

It was observed that excess Na^+^ was adsorbed onto Mg(OH)_2_ particles, reducing lithium entrapment in the solid phase and enabling high lithium recoveries between 96% and 99% in the remaining aqueous solution. Conversely, when the concentration of Li^+^ predominated over that of Na^+^, lithium recovery decreased to 77–81% due to greater lithium retention in the precipitated solids. It is important to note that some critical variables, such as temperature and flow rate, were not varied in the study, which limited the comprehensive characterization of the system’s performance.

Although promising results were obtained, the challenge of improving lithium recovery under low-impurity conditions remains, particularly in solutions obtained from DLE processes where lithium is the predominant cation in solution [2]. A relevant approach to address this challenge is to control the nucleation and growth rate of Mg(OH)_2_ particles, which is influenced by current density and temperature [19,20]. High current densities generate more OH^−^, promoting local supersaturation and rapid nucleation, while low current densities can favor a more controlled particle growth, reducing aggregate formation and improving the purity of the precipitate. On the other hand, temperature increases ionic mobility, helping to reduce energy consumption, and it influences the nucleation and growth of Mg(OH)_2_ crystals [21]. The synthesis temperature directly affects the morphology of Mg(OH)_2_: at 10 °C, nucleation is slow, resulting in a few large particles; at 45 °C, individual nanosheets with controlled size are formed; and above 65 °C, rapid nucleation leads to aggregation and the formation of flower-like structures [22,23].

The nucleation of Mg(OH)_2_ particles occurs once supersaturation is reached. This process depends on the flow rate [24], degree of saturation, and the presence of impurities [25]. High flow rates enhance mixing and promote the formation of small nuclei, while low flow rates allow for more defined crystal growth due to increased residence time [26]. Greater supersaturation increases nucleation, which is undesirable when aiming to control particle size.

When using precipitating agents, temperature plays a key role in Mg(OH)_2_ particles growth, with higher temperatures favoring crystal nucleation and promoting particle formation [27]. In contrast, low flow rates increase the residence time and promote the formation of larger particles [25,26,27].

An important aspect of the purification process is the interaction between precipitated particles and dissolved ions. These interactions, which depend on both the particle size and the surface charge of the precipitates, may result in unwanted lithium loss due to brine entrapment within the aggregated solids or via adsorption [28,29]. In this context, the zeta potential, which reflects the effective surface charge of particles, is a key parameter.

The surface charge and zeta potential influence the adsorption of cations such as Ca^2+^, Na^+^, and K^+^ onto Mg(OH)_2_ particles [28,29]. These parameters depend on pH, particle size, and ionic strength [30]. Smaller particles have a higher surface charge, which favors adsorption, while larger particles exhibit lower adsorption capacity. Likewise, a high concentration of impurities compresses the electric double layer, reducing the zeta potential and shifting the isoelectric point (IEP) [29,31]. Conversely, in solutions with lower ionic strength, the electrical double layer extends, enhancing the effect of surface charge. Thus, understanding the surface properties of Mg(OH)_2_ and Ca(OH)_2_ may be considered essential for optimizing lithium recovery, reducing brine entrapment, and lithium adsorption.

Temperature also affects zeta potential, influencing both the colloidal particle properties and the surrounding medium. In particular, it modifies parameters such as solvent’s dielectric constant, ion mobility, thickness of the diffuse layer, colloidal stability, and the viscosity of the medium. Consequently, dissolution–precipitation processes at the mineral–solution interface govern the surface charge and zeta potential value [32].

The isoelectric point corresponds to the pH at which the particle surface has no net charge (IEP = 0) [29,30,31,32,33,34]. Under these conditions, only gravitational forces act between particles, leading to agglomeration. The further the system moves away from the isoelectric point, the more stable the system becomes. In general, the boundary between a stable and unstable suspension is considered to be around ±30 mV [35,36]. The higher the absolute value of zeta potential, the more likely the suspension is to remain stable as the charged particles will repel each other overcoming their natural tendency to agglomerate [37].

Based on the limitations identified in previous studies, this work proposes a modification of the three-compartment electrodialysis system by adopting a different approach. In the new setup, H_2_SO_4_ is introduced into the anolyte, generating H^+^ ions that migrate toward the central compartment, where HCl is concentrated. This HCl is enriched by the migration of Cl^−^ from the catholyte and H^+^ from the anolyte through commercial selective membranes (CMX and AMX Neosepta). In the cathodic compartment, a LiCl-rich brine containing impurities (Mg^2+^, Ca^2+^, Na^+^, and K^+^) is used. In this compartment, the reduction of water generates OH^−^ ions, which rapidly react with the divalent cations to form their respective hydroxide precipitates. Unlike previous studies, the aim is not to recover a pure Mg(OH)_2_ solid but to achieve efficient impurity removal that maximizes lithium recovery in the treated brine. In parallel, the process generates co-products such as HCl and H_2_SO_4_, which are widely used in the copper industry and other productive sectors.

To optimize impurity removal and enhance Li^+^ recovery, it is essential to understand the physicochemical phenomena occurring in the catholyte. The interaction between OH^−^ ions and divalent cations triggers a process of supersaturation, nucleation, and particle growth. These particles develop a surface charge and a zeta potential [29], the magnitude of which depends on pH and directly affects their colloidal stability, hydrodynamic size, and tendency to aggregate or remain dispersed.

Although zeta potential is related to surface charge, it does not directly represent it. Instead, it reflects the electrical potential at the slip plane within the electric double layer, which depends on the ion distribution surrounding the particle. This distinction is crucial to understanding how operational conditions can be tuned to favor the formation of larger particles with surface properties that minimize cations losses such Li^+^, thereby facilitating solid–liquid separation by filtration with minimal lithium loss.

The aim of this study is to evaluate the effect of temperature (13.8–56 °C) and current density on the removal of Mg^2+^ and Ca^2+^, as well as on the zeta potential of the resulting particles. These parameters are used to identify an operational window that promotes the formation of Mg(OH)_2_ particles with favorable physicochemical properties, specifically particles that are easily separable and have a surface charge that could reduce Li^+^ losses, thus enhancing lithium recovery from LiCl brines.

In all experiments, a constant high flow rate is maintained to prevent stagnant zones and reduce the risk of solid deposition, consistent with previous findings [18,24,28]. Current density is selected based on the amount of OH^−^ required to reach a basic pH sufficient to precipitate Mg(OH)_2_ from solution. Low-to-moderate current densities are applied since high values may increase energy consumption and promote excessive gas formation, negatively impacting system efficiency [2].

This configuration enables the simultaneous removal of Mg^2+^ and Ca^2+^ without using chemical precipitating agents, producing only H_2_, O_2_, and HCl as by-products, and avoiding Cl_2_ formation, which significantly reduces the environmental impact.

This study addresses the membrane-assisted electrochemical removal of Mg^2+^ and Ca^2+^ by analyzing the evolution of the zeta potential of Mg(OH)_2_ and Ca(OH)_2_ particles precipitated in an electrodialysis system operated under controlled temperature and current density conditions, an aspect that has been scarcely explored in the literature. A wide temperature range was evaluated, including 13.8 °C, which is below ambient temperature. In addition, current densities were carefully adjusted to ensure that the molar generation rate of OH^−^ remained below the molar flow rate of recirculated Mg^2+^ in order to avoid local supersaturation and allow for controlled particle growth.

## 2. Materials and Methods

### 2.1. Reagents and Materials

A synthetic solution was prepared to represent a lithium-rich brine containing impurities. For this purpose, high-purity reagents from Merck Millipore (Billerica, MA, USA) were used, including hydrochloric acid (HCl, 37%), sulfuric acid (H_2_SO_4_, 98%), lithium chloride (LiCl, ≥99%), potassium chloride (KCl, ≥99%), sodium chloride (NaCl, ≥99%), magnesium chloride hexahydrate (MgCl_2_·6H_2_O, ≥99%), and calcium chloride dihydrate (CaCl_2_·2H_2_O, ≥99%). The synthetic lithium solution simulates a concentrated brine with a high lithium content and low concentrations of impurities, consistent with brine obtained through direct lithium extraction (DLE) processes, which typically exhibit low pH (~1.2) due to previous desorption steps. The chemical composition of the initial synthetic brine is shown in Table 1, along with the concentration ratios of lithium over other cations. These ratios represent the proportion of lithium concentration compared to each impurity and are used as indicators of selectivity and purification demand.

### 2.2. Cell Design and Membrane Electrodialysis System

**Figure 1:**
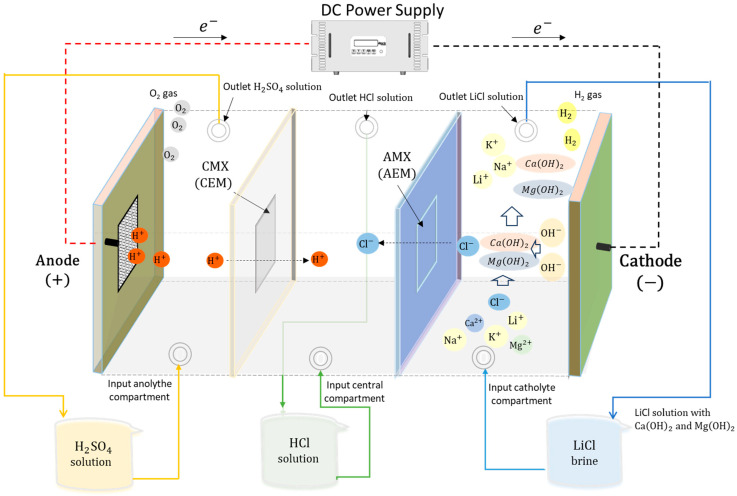
Schematic of the electrodialysis cell with three compartments. CEM: cation exchange membrane; AEM: anion exchange membrane. Solid arrows indicate electrolyte flow (blue, green, yellow), and dashed lines indicate electric current (red, black).

The experimental system consisted of a membrane electrolysis reactor with three compartments, separated by a Neosepta AMX anion-exchange membrane and a Neosepta CMX cation-exchange membrane (ASTOM Corporation, Tokyo, Japan), both with an effective area of 4 cm^2^ (see Figure 1). The electrodialysis cell was fabricated from PTFE, featuring a rectangular geometry and an open-to-atmosphere design, with electrolyte recirculation driven by gravity. Each compartment has a volume of 52 cm^3^.

Electrolysis was carried out using a direct current power supply provided by a rectifier (model GPR-1810HD, GW Instek, New Taipei, Taiwan), and the flow velocity was controlled at 0.808 cm/s in the compartments using peristaltic pumps (model 520SN/R2, Watson-Marlow, Falmouth, UK). Temperature and pH were measured using a multimeter (model HI5522, Hanna Instruments, Smithfield, RI, USA).

The generation of OH^−^ ions in the cathode compartment occurs through the reduction of water, which subsequently reacts with the Mg^2+^ and Ca^2+^ cations present in the LiCl solution, forming Mg(OH)_2_ and Ca(OH)_2_ precipitates according to Equations (1) and (2), respectively.

(1) $${\mathrm{Mg}}_{\left(\mathrm{ac}\right)}^{2+}+2{\mathrm{OH}}_{\left(\mathrm{ac}\right)}^{-}↔{\mathrm{Mg}\left(\mathrm{OH}\right)}_{2\;\left(s\right)}$$

(2) $${\mathrm{Ca}}_{\left(\mathrm{ac}\right)}^{2+}+2{\mathrm{OH}}_{\left(\mathrm{ac}\right)}^{-}↔{\mathrm{Ca}\left(\mathrm{OH}\right)}_{2\;\left(s\right)}$$

In the anolyte compartment, 150 g of 0.5 M H_2_SO_4_ was recirculated, generating protons (H^+^) and oxygen gas via water oxidation, i.e., the anodic reaction: 2H_2_O → O_2_ + 4H^+^ + 4e^−^. The H^+^ ions migrate to the central compartment through the CMX cation exchange membrane. In the central compartment, 150 g of 0.5 M HCl was added, which gradually increases in concentration due to the migration of Cl^−^ from the catholyte and H^+^ from the anolyte.

The study focused on the cathodic compartment. Ten tests were conducted using the synthetic solution, following a central composite design with star configuration and randomization of the trial order, according to the response surface methodology. The experimental design is presented in Table 2.

pH was measured every 10 min. From pH 8 to 12.5, 3 mL aliquots were collected at each pH unit for zeta potential analysis using a Litesizer 501 instrument (Anton Paar GmbH, Graz, Austria). This measurement is essential for identifying the isoelectric point and evaluating the colloidal stability of the precipitates formed. At the same time, cell voltage and electric current were recorded over time. The current density was selected based on the OH^−^ required to reach a basic pH enabling Mg(OH)_2_ precipitation. Operating time varied depending on the conditions and was stopped either when a final pH of 12 was reached to ensure complete removal of Mg^2+^ or when the pH stabilized to avoid unnecessary energy consumption.

At the end of each experiment, the catholyte was filtered and analyzed to determine the final concentrations of Li^+^, Na^+^, K^+^, Ca^2+^, and Mg^2+^. The concentrations of sodium, potassium, calcium, lithium, and magnesium were determined by atomic absorption spectrometry, while chloride was determined by volumetric titration with AgNO_3_.

The specific electrical energy consumption (SEC) per mass of lithium recovered is calculated using Equation (3).

(3) $$SEC=\int_{0}^{t}\frac{E\cdot I}{m_{Li}}dt$$

Here, *E* is the voltage drop across the cell (V), *I* is the applied current (A), and $m_{Li}$ is the mass of recovered lithium (kg).

The removal percentage of Mg^2+^ and Ca^2+^ was calculated using Equation (4):

(4) $$\%Removal=\;\left(\frac{C_{o}M_{o}-C_{f}M_{f}}{C_{o}M_{o}}\right)*100\%$$

Here, *C*_*o*_ and *C*_*f*_ are the initial and final concentrations of the substance (% mass), and *M*_*o*_ and *M*_*f*_ correspond to the initial and final mass of the catholyte (g).

The lithium recovery percentage was calculated using Equation (5).

(5) $$\%Recovery=\frac{C_{Lif}M_{f}}{C_{Lio}M_{o}}*100\%$$

Here, *C*_*Lio*_ and *C*_*Lif*_ are the initial and final lithium concentrations in the catholyte (% mass), while *M*_*o*_ and *M*_*f*_ are the initial and final mass of the catholyte (g).

Theoretical OH^−^ generation was estimated using Faraday’s law, assuming that all the applied electric charge was used for water reduction at the cathode. The charge (Q) was converted to moles of OH^−^ ($n_{OH^{-}}$) using Equation (6), where F is Faraday’s constant (96,485 C/mol).

(6) $$n_{OH^{-}}=\frac{Q}{F}$$

The apparent faradaic efficiency for OH^−^ utilization was then calculated by comparing the theoretical OH^−^ generated with the actual OH^−^ consumed in the neutralization of the initial brine and in the experimentally quantified OH^−^ incorporated into the formed Mg(OH)_2_ and Ca(OH)_2_ solids.

### 2.3. Statistical Analysis

To assess the performance of the developed set of experiments, several statistical indicators were used to evaluate their explanatory power and the validity of their predictions.

The coefficient of determination ($R^{2}$) quantifies the proportion of the total variability in the dependent variables that are explained by the set of experiments. It was calculated according to the Equation (7) [38,39].

(7) $$R^{2}=1-\frac{SSE}{SST}$$

where *SSE* is the sum of squared errors (residuals), and *SST* is the total sum of squares (total variability of the data).

The adjusted coefficient of determination ($R_{adjusted}^{2}$) was employed to correct $R^{2}$ based on the number of predictors included in the model, penalizing the inclusion of irrelevant variables. It is defined according to Equation (8).

(8) $$R_{adjusted}^{2}=1-\left(\frac{\left(1-R^{2}\right)\left(n-1\right)}{n-p-1}\right)$$

where *n* is the number of observations, and *p* is the number of independent variables.

The standard error of the estimate (S) measures the average deviation between the observed and predicted values. It was calculated using Equation (9).

(9) $$S=\sqrt{\frac{\sum {\left(y_{i}-\hat{y_{i}}\right)}^{2}}{n-p-1}}$$

where *y_i_* are the observed values, *ŷ_i_* are the predicted values, *n* is the number of observations, and *p* is the number of predictors.

The analyses were performed using Statgraphics version 19.4.04 (Statgraphics Technologies, Inc., The Plains, VA, USA) with a significance level of 5% (α = 0.05).

## 3. Results and Discussion

### 3.1. pH Evolution and Removal of Mg^2+^ and Ca^2+^

**Figure 2:**
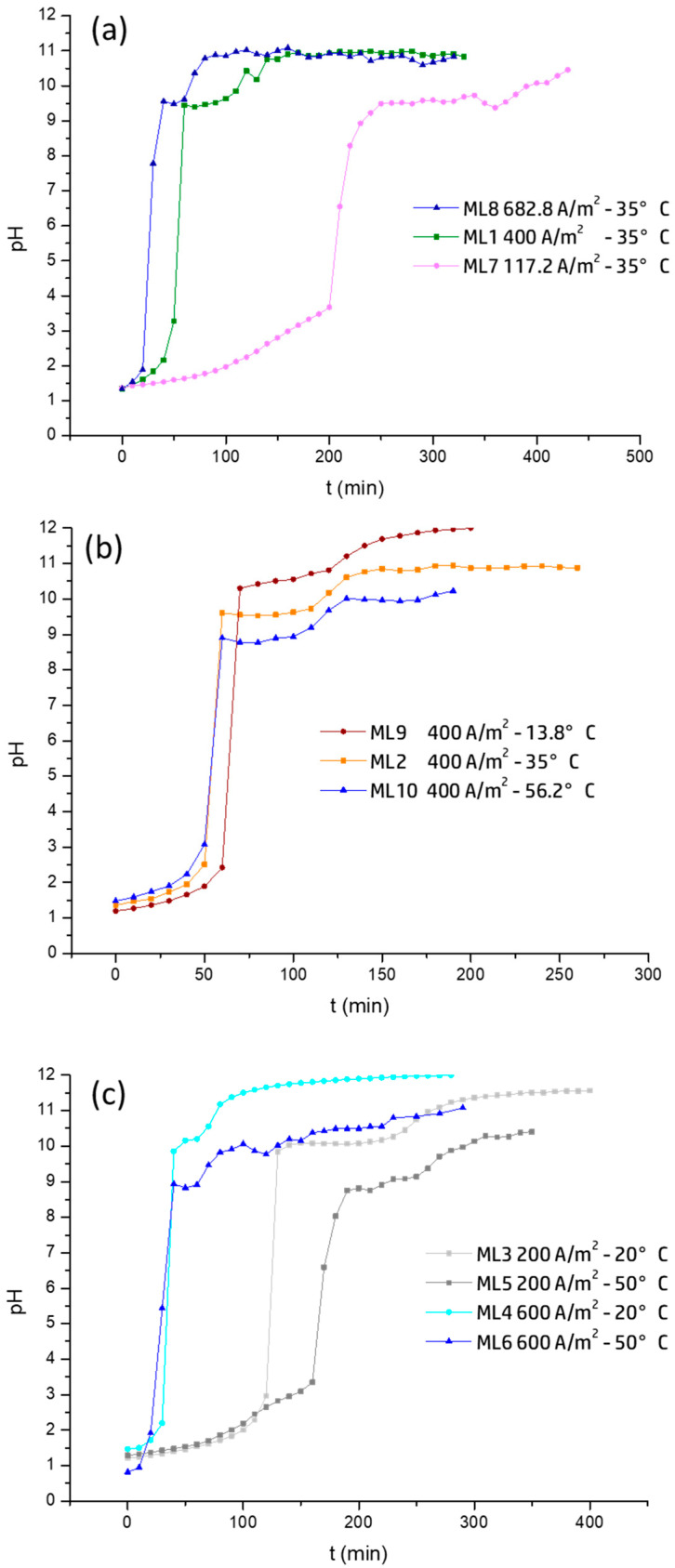
pH variation over time. (**a**) Effect of current density; (**b**) effect of temperature; (**c**) combined effect of current density and temperature.

Figure 2 highlights the distinct pH plateaus that correspond to the sequential precipitation of Mg(OH)_2_ and Ca(OH)_2_, enabling the identification of the onset and extent of each precipitation step. Figure 2a shows the variation of pH in the catholyte for the different current densities at 35 °C. Mg(OH)_2_ begins to precipitate at approximately pH 9.5, while Ca(OH)_2_ precipitation occurs near pH 11. Notably, the pH values at which precipitation starts remain constant across all current densities. This behavior is consistent with the thermodynamic solubility limits (Ksp) of each hydroxide and suggests that equilibrium conditions govern the precipitation thresholds rather than the OH^−^ production rate. In other words, each hydroxide precipitates when the OH^−^ concentration reaches the level dictated by its solubility product (Ksp), regardless of how that OH^−^ is generated.

Therefore, while the current density accelerates the pH increase and reduces the time to reach precipitation, it does not affect the precipitation pH itself. This distinction is important as it indicates that the system’s driving force for precipitation is thermodynamic, not kinetic [40]. Thus, the invariance of the precipitation pH is not trivial but rather highlights the independence of saturation conditions from operational parameters such as the current density. It is important to clarify that the constancy of the precipitation pH does not imply a constant zeta potential. Even though the pH at which the Mg(OH)_2_ and Ca(OH)_2_ precipitate remains stable under fixed temperature conditions, the zeta potential can still vary due to factors such as ionic strength, electrolyte composition, or the evolving surface properties of the precipitating solids.

**Figure 3:**
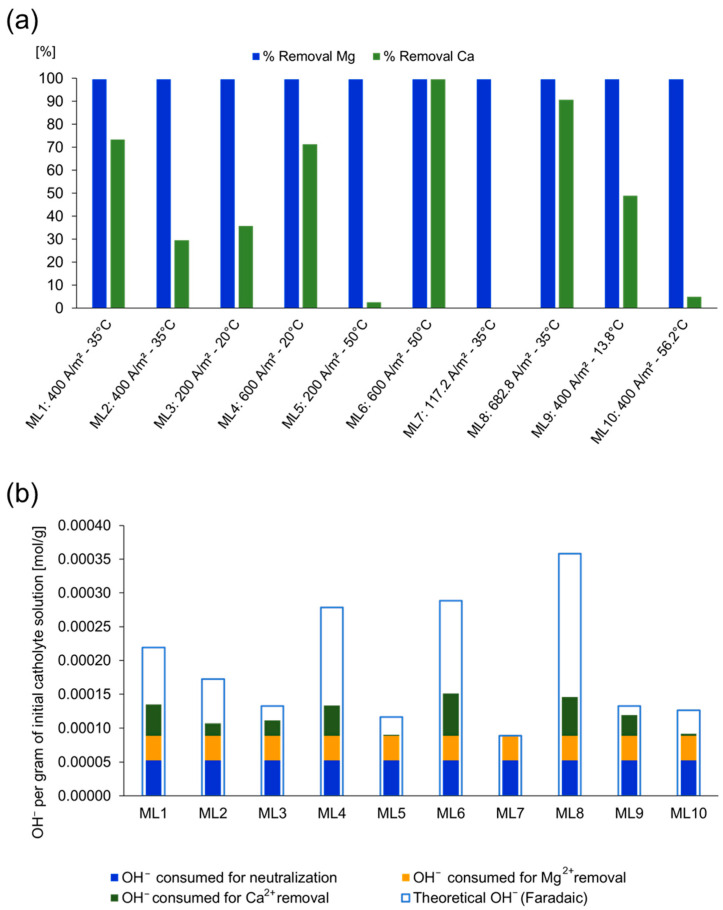
Magnitude of electric charge applied in each experimental test. (**a**) Removal of Mg and Ca; (**b**) OH^−^ utilization vs. theoretical generation based on applied charge.

When OH^−^ generation is insufficient to reach the thermodynamic threshold for Ca(OH)_2_ precipitation, as observed in ML7 with a current density of 117.16 A/m^2^, only the pH plateau corresponding to magnesium precipitation was observed, with no indication of a second pH plateau corresponding to calcium removal. This indicates that the OH^−^ produced under those conditions was not sufficient to reach the pH required for Ca(OH)_2_ precipitation, thus limiting calcium removal. This is confirmed by the low Ca^2+^ removal observed in Figure 3a.

Figure 2b shows the influence of temperature on pH evolution. It can be observed that the apparent solubility product (pKps) is strongly affected by temperature. At 13.8 °C (ML9), the precipitation of Mg(OH)_2_ occurs around a pH of 10.5, while at 35 °C (ML2), the pH decreases to 9.6, and at 56.2 °C (ML10), it drops further to pH 8.6. This trend indicates that as the temperature increases, the pH required to initiate precipitation decreases. This behavior is explained by the fact that increasing the temperature enhances ion collisions and accelerates the kinetics of precipitation. As a result, the precipitation reaction is thermodynamically more favorable [41,42]. Heat promotes the formation of the solid according to the Van’t Hoff equation, which describes how temperature affects the equilibrium constant of the reaction. This raises the equilibrium constant (Kps) and lowers the Gibbs free energy (ΔG°), making the reaction more spontaneous. As a result, the solubility product (Ksp) increases, and a lower concentration of OH^−^ is required for precipitation, causing the precipitation pH to decrease with temperature [41]. At low temperatures, the high viscosity of the electrolyte and the low ionic mobility hinder the interaction between Mg^2+^ and OH^−^, delaying the formation and detachment of Mg(OH)_2_, thus reducing the current efficiency. Since the dissolution of Mg(OH)_2_ is endothermic, an increase in temperature favors solubility. At the same time, higher temperature enhances ionic activity and reduces viscosity, facilitating ion collisions and accelerating the kinetics of precipitation [43]. Therefore, temperature not only affects the rate of solid formation but also alters the chemical equilibrium between the dissolved phase and the precipitate, thereby modifying the effective value of the pKps. Figure 2b shows that at a current density 400 A/m^2^, all three temperatures evaluated (13.8 °C, 35 °C, and 56.2 °C) allow for the appearance of a second pH plateau associated with the precipitation of Ca(OH)_2_. Interestingly, among these tests (ML2, ML9, and ML10), the system achieved the highest calcium removal efficiency at the lowest temperature (13.8 °C), while magnesium removal remains close to 99% in all cases.

Figure 2c summarizes the combined effects of current density and temperature on pH variation. At 20 °C, both test ML3 and ML4 reach higher final pH values and show a clear plateau attributed to calcium precipitation. This confirms that lower temperature favors higher pH, while a higher current density reduces the time to reach precipitation, without necessarily increasing the final pH.

Theoretical models indicate that higher current density leads to increased nucleation, generating smaller particles [19,44]. This increases the consumption of OH^−^ in forming Ca(OH)_2_, preventing further pH rise. In contrast, higher temperature delays precipitation, allowing OH^−^ to accumulate in solution and increase pH.

For test ML5, a delayed onset of the first pH plateau was observed (Figure 2c), indicating slower Mg(OH)_2_ formation. In general, magnesium removal was nearly complete in all tests in line with the objective of the experimental design, which aimed to reach a final pH of 12. However, this pH was not always reached, and calcium removal varied depending on the total electrical charge applied, which determined OH^−^ production (Figure 3b).

Figure 3b shows the difference between the theoretical OH^−^ generated (calculated from the applied electric charge) and the actual OH^−^ consumed for Mg^2+^ and Ca^2+^ removal and acid neutralization. This difference provides an indirect measure of the faradaic efficiency for OH^−^ utilization. A smaller gap suggests a more efficient use of the generated OH^−^, while a larger gap indicates losses due to side reactions, limitations of mass transport, or incomplete mixing. The apparent faradaic efficiency for OH^−^ utilization appears to be highly influenced by the efficiency of Ca^2+^ removal. In test ML7, where no Ca^2+^ removal was observed, the OH^−^ consumed for acid neutralization and Mg^2+^ removal was nearly equivalent to the theoretical OH^−^ generated, suggesting a high efficiency in Mg^2+^ removal under the tested conditions. In contrast, test ML6 and ML8, which achieved higher Ca^2+^ removal, showed larger gaps compared to theoretical OH^−^, indicating lower efficiency. This reduced efficiency in current utilization is attributed to poor mixing within the cell caused by the accumulation of Mg(OH)_2_ solids, which can hinder mixing and mass transport. Additionally, possible side reactions may further contribute to OH^−^ consumption losses. Moreover, the application of an electric charge exceeding the stoichiometric requirement for complete precipitation leads to an overproduction of OH^−^, which increases energy consumption without enhancing removal performance. Together, these factors suggest the existence of an optimal applied charge that balances efficiency and selectivity.

The best overall removal of both Mg^2+^ and Ca^2+^ was achieved in test ML6 by operating at 600 A/m^2^ and 50 °C, indicating that these parameters interact to favor Ca(OH)_2_ nucleation. However, its lower apparent faradaic efficiency indicates that the process was not optimal in terms of energy use. A slightly lower charge (ML4) failed to fully remove Ca^2+^, while a higher charge (ML8) led to increased energy consumption without improving removal performance. Notably, test ML9 (13.8 °C) exhibited high apparent faradaic efficiency, suggesting that operating at lower temperatures can improve OH^−^ utilization. This may be attributed to slower precipitation kinetics, which favor more controlled particle growth. Future research should focus on identifying the optimal applied electrical charge and implementing a two-stage removal strategy for Mg^2+^ and Ca^2+^. This approach could minimize the hindering effect caused by early Mg(OH)_2_ precipitation, improve faradaic efficiency, and facilitate selective separation.

### 3.2. Zeta Potential Evolution During Electrochemical Precipitation

The zeta potential, which reflects the electrical potential at the slipping plane of suspended particles [29], determines whether particles tend to repel each other or aggregate in the liquid. This aggregation behavior, in turn, influences the removal of Mg^2+^ and Ca^2+^ and ultimately affects lithium recovery.

All zeta potential values were measured in triplicate, with standard deviation below ±1.08 mV, confirming the reproducibility of the data.

**Figure 4:**
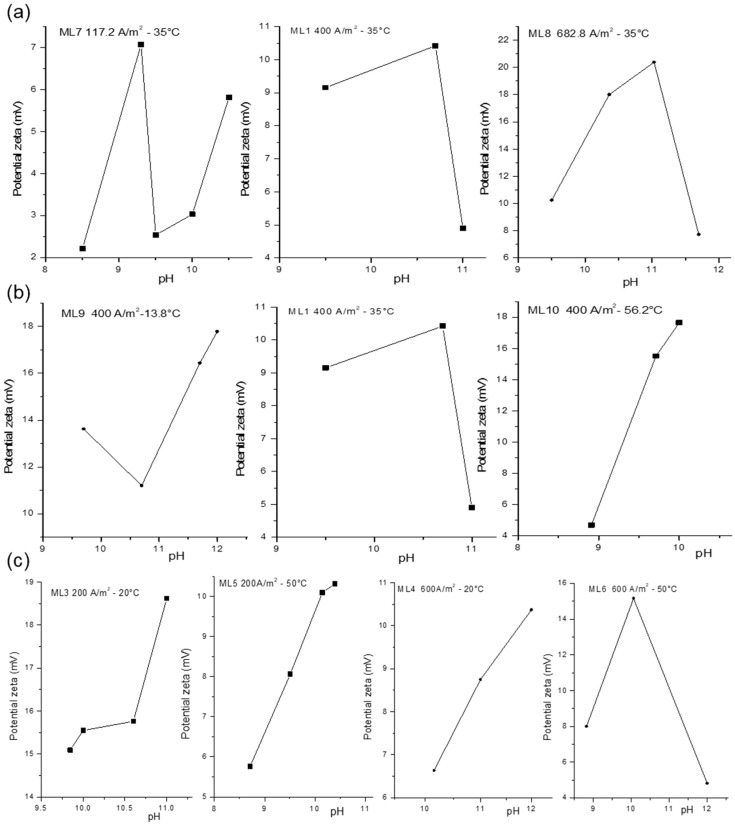
Zeta potential vs. pH: (**a**) influence of current density; (**b**) influence of temperature; (**c**) influence of temperature and current density.

Figure 4 shows the measured zeta potential values as a function of the pH for particles formed under different temperatures and current densities. In all cases, the precipitated particles exhibited positive zeta potential values, ranging from +4 to +20 mV. This positive surface charge generates electrostatic repulsion with the anion-exchange membrane, which likely contributed to the absence of membrane fouling, as previously reported by Breite et al. [45] and Abdelrasoul et al. [46]. In contrast, particle accumulation was observed at the cathode surface, likely due to electrostatic attraction between the positively charged particles and the negatively charged electrode.

The trends in the zeta potential correspond with the plateaus identified in the pH evolution profiles (Figure 2) and to the observed Mg^2+^ and Ca^2+^ removal results (Figure 3a). In general, for each graph in Figure 4, the first measured zeta potential value corresponds to the point immediately after neutralization of the acidic brine, marking the onset of Mg(OH)_2_ precipitation. As precipitation proceeds, a rise in zeta potential is observed, which may be attributed to reduced availability of free OH^−^ ions and changes in the electric double layer, probably due to the depletion of OH^−^ ions in solution as Mg(OH)_2_ particles form. Consequently, the maximum value of the zeta potential coincides with the pH at which Mg^2+^ precipitation ends, followed by an increase in pH and the beginning of Ca(OH)_2_ precipitation, evidenced by a second pH plateau. Finally, the formation of Ca(OH)_2_ leads to a decrease in zeta potential due to the continuous depletion of OH^−^ in the solution.

#### 3.2.1. Influence of Current Density on Zeta Potential

Figure 4a shows the variation of zeta potential at three different current densities at a temperature of 35 °C. In all cases, it can be observed that as the pH increases, the zeta potential also rises until reaching a maximum value, which increases with both pH and the applied current density. The maximum zeta potential values obtained for current densities of 117, 400, and 682 A/m^2^ were 7.3, 10.7, and 20.03 mV, respectively. This trend may be attributed to the formation of smaller particles with higher specific surface area at higher current densities, which enhances the development of surface charge and thus increases the zeta potential.

In test ML7, which used the lowest current density, the first measure of zeta potential was recorded after brine neutralization and the formation of initial Mg(OH)_2_ particles. The zeta potential then increased until it reached its maximum value at a pH of 9.3 before decreasing. This decline coincided with the prolonged pH plateau associated with Mg(OH)_2_ precipitation (see Figure 2a). The slow pH change indicates a low precipitation rate of Mg(OH)_2_ implying that the rate of OH^−^ generation at the cathode exceeds that of particle formation. This resulted in an excess of OH^−^ ions around the positively charged Mg(OH)_2_ particles, causing a decrease in the zeta potential [47].

After reaching a pH of 9.5, the zeta potential increases again, possibly indicating OH^−^ consumption as Ca(OH)_2_ began to precipitate. However, in test ML7, the second pH plateau was not observed, indicating negligible Ca(OH)_2_ formation, which is supported by the absence of calcium removal in this test (see Figure 3a).

In test ML1 (at 400 A/m^2^), the first particles were formed at a pH close to 9.3. The initial pH plateau associated with Mg(OH)_2_ formation is short, suggesting rapid precipitation, which is reflected in the increase in zeta potential up to a pH of 10.7 (Figure 2a). The second pH plateau, associated with Ca(OH)_2_ precipitation, was accompanied by a gradual decline in zeta potential, likely due to the depletion of OH^−^ in solution. It can be deduced that, as this pH plateau extends over time, the zeta potential tends to decrease. This correlates with a significant calcium removal efficiency of 73%.

In test ML8, the first measure of zeta potential at pH 9.5 coincides with the onset of the first pH plateau observed in Figure 2a. Due to the high current density, OH^−^ was rapidly generated, resulting in a brief plateau and a sharp increase in zeta potential at pH 11.03. As Ca(OH)_2_ precipitation progressed and OH^−^ was consumed, the zeta potential decreases again. This test achieved a calcium removal efficiency of 90.7%.

Although the experimental conditions did not reach the isoelectric point (IEP), the measured zeta potential remained positive and exhibited a clear decreasing trend with a pH increase near to 12. This behavior is consistent with electrostatic theory, which predicts a gradual decrease in surface charge as the pH increases and approaches the isoelectric point (IEP) from lower pH values [23,28,48].

#### 3.2.2. Influence of Temperature on Zeta Potential

Figure 4b shows the variation of the zeta potential with temperature at a constant current density of 400 A/m^2^, which can be complementarily analyzed with Figure 2b. At a temperature of 13.8 °C (ML9), the zeta potential initially decreases at pH 10.7, followed by an increase up to a pH of 12. This behavior is associated with the progression of Mg(OH)_2_ precipitation. Once magnesium precipitation is completed, excess OH^−^ ions may accumulate in solution, potentially reducing surface charge due to ionic shielding, which contributes to the observed decrease in the zeta potential at pH 10.7. The decrease in zeta potential after Mg(OH)_2_ precipitation is notable at a low temperature where precipitation kinetics are slower [21]. The increase in pH beyond 10.7 is attributed to the transition toward Ca(OH)_2_ precipitation. In this test, however, no further decrease in zeta potential is observed, consistent with the low Ca^2+^ removal efficiency reported for ML9 (see Figure 3a). This suggests limited formation of calcium precipitate.

For test ML1 at 35 °C, a more extended pH plateau corresponding to Ca(OH)_2_ precipitation is observed, resulting in slower precipitation and, therefore, a decrease in zeta potential. On the other hand, for test ML10 at 56.2 °C, the higher ionic mobility accelerates nucleation, and an increase in zeta potential is observed up to values of 17.6 mV at pH 10, without a subsequent decrease. This behavior correlates with the limited Ca^2+^ removal (5%) and the relatively low final pH attained in this test.

In both ML5 and ML10, the insufficient electric charge was calculated based only on the stoichiometric requirement for Mg^2+^ removal, which proved inadequate for complete Ca^2+^ precipitation (see Figure 3b).

Figure 4c illustrates the combined effects of temperature and current density. At 600 A/m^2^, increasing the temperature changes the final zeta potential value obtained. For test ML6 at 600 A/m^2^ and 50 °C, the final zeta potential reached is the minimum of 4.8 mV at pH 12, coinciding with the highest observed Ca^2+^ removal of 99.5%.

### 3.3. Recovery of Lithium, Sodium, and Potassium and Specific Electrical Energy Consumption

Zeta potential measurements showed that the surface charge never exceeded 20.3 mV. This level of charge is not expected to induce strong electrostatic repulsion against monovalent cations in solution. Since zeta potential primarily reflects colloidal stability rather than direct adsorption affinity, this limited surface charge could favor the entrapment of brine within the precipitate.

**Figure 5:**
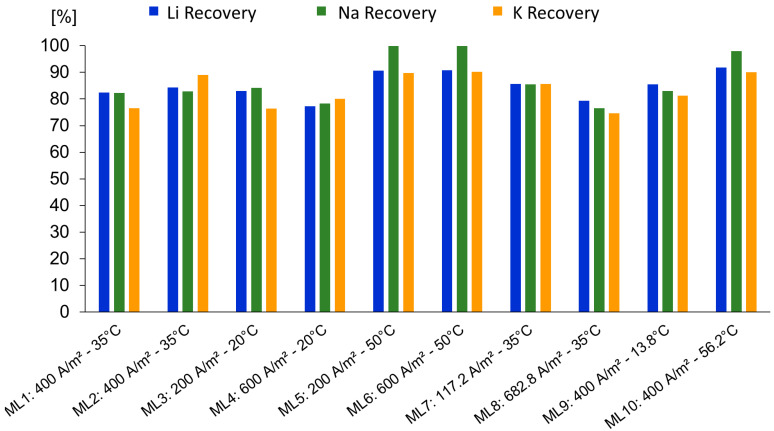
Recovery of Li, Na, and K for each experimental test.

As shown in Figure 5, the highest recovery was observed for Na^+^, followed by Li^+^ and K^+^. As expected, the predominant ion exhibits the highest loss due to the entrapment of brine within the precipitated solids [2].

Tests ML5, ML6, and ML10, conducted at 50 and 56.2 °C, showed the highest recoveries of lithium, sodium, and potassium, with values of 90.7%, 90.8%, and 91.8%, respectively. Among these, only test ML6 also achieved high calcium removal (99.5%), while ML5 and ML10 showed significantly lower Ca^2+^ removals (2.5% and 5.0%, respectively). The corresponding zeta potential values for ML6, ML5, and ML10 were 4.8 mV, 10.3 mV, and 17.6 mV, measured at final pH values of 12, 10.4, and 10, respectively. This result highlights the correlation between lower zeta potential values and enhanced Ca^2+^ removal, indicating that approaching the isoelectric condition at pH 12 promotes selective precipitation of divalent impurities while sustaining high lithium recoveries.

Zeta potential does not directly quantify ion adsorption but reflects the net charge at the slipping plane, which may influence adsorption dynamics. For tests ML4 and ML9, although pH 12 was reached, only a partial calcium removal of 71% and 49% was achieved, with zeta potential values of 10.4 mV and 17.8 mV, respectively. These differences can be more directly attributed to variations in applied charge rather than surface charge. Therefore, improving lithium recovery and calcium removal requires a combined strategy optimizing applied current and time to complete the precipitation. This approach could also contribute to reduced lithium co-removal through improved control of surface charge and aggregation conditions.

**Figure 6:**
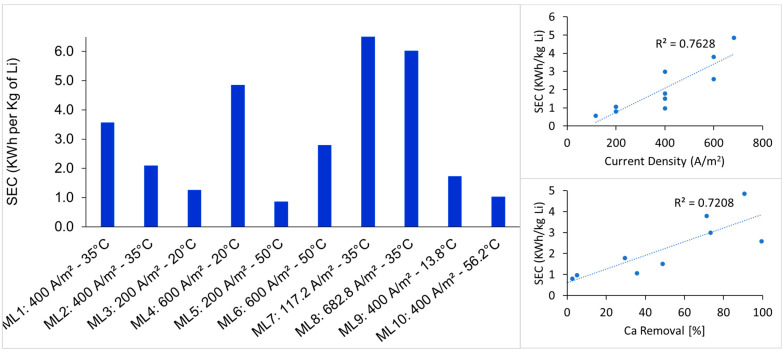
Specific electrical energy consumption per kilogram of lithium recovered from the brine for each experimental run.

Figure 6 shows the specific electrical energy consumption for each experimental test per kilogram of lithium recovered. As expected, SEC increased with both current density and calcium removal. However, higher operating temperatures resulted in lower SEC at comparable current densities, likely due to improved ion mobility and precipitation kinetics.

The best overall result for the removal of Ca^2+^ and Mg^2+^, as well as Li recovery, was obtained in test ML6, which required 2.58 kWh per kg of lithium recovery. This value is significantly lower than previously reported values (20.28–34 Kwh/kg) [2] even under similar chemical compositions. This improved efficiency can be attributed to the combination of moderate current density, elevated temperature, and the specific cell design, which may improve mixing and reduce resistive losses. However, despite the favorable removal and recovery performance, test ML6 exhibited a low faradaic efficiency, suggesting energy losses due to excess charge input, potentially associated with overgeneration of OH^−^.

### 3.4. Chemical Composition of the Final Brine

This study demonstrates that electrochemical precipitation, although established in principle, can be effectively applied under mild current densities and moderate temperatures, achieving removal of divalent cations, enhanced lithium recovery, and co-generation of hydrochloric acid, without adding external chemical reagents.

Table 3 summarizes the ionic ratios in the final brine. The Li/Mg and Li/Ca ratios were drastically improved, especially in test ML6, where the Li/Mg ratio increased from 26.36 to 7850 and Li/Ca from 9.21 to 2617.

The Li/Na and Li/K ratios remained relatively stable, indicating that alkali metal ions were not affected by the precipitation process. This suggests a high degree of selectivity for the removal of cations such Mg^2+^ and Ca^2+^.

Additionally, in the tests that showed the best results, the final concentrations of Li^+^, Na^+^, and K^+^ in catholyte were higher than the initial values. This can be attributed to a volume reduction caused by selective electroosmosis [49,50] and hydroxide-driven precipitation, leading to a concentration effect. Such behavior is characteristic of divided electrochemical cells, where ion-selective membranes facilitate both ionic separation and concentration.

Furthermore, the migration of Cl^−^ ions across the Neosepta AMX anion exchange not only supports electroneutrality but also enables the in situ generation of HCl in the central compartment. In test ML6, this resulted in an HCl concentration of 3.8 M, representing a valuable by-product that enhances the sustainability of the process by reducing the need for external chemical inputs.

In summary, this work advances the conventional framework of electrochemical precipitation by demonstrating a selective, energy-efficient, and scalable route for removing Mg^2+^ and Ca^2+^ from lithium-rich brines while simultaneously concentrating lithium and producing HCl as a co-product.

### 3.5. Analysis of Response Surfaces

**Figure 7:**
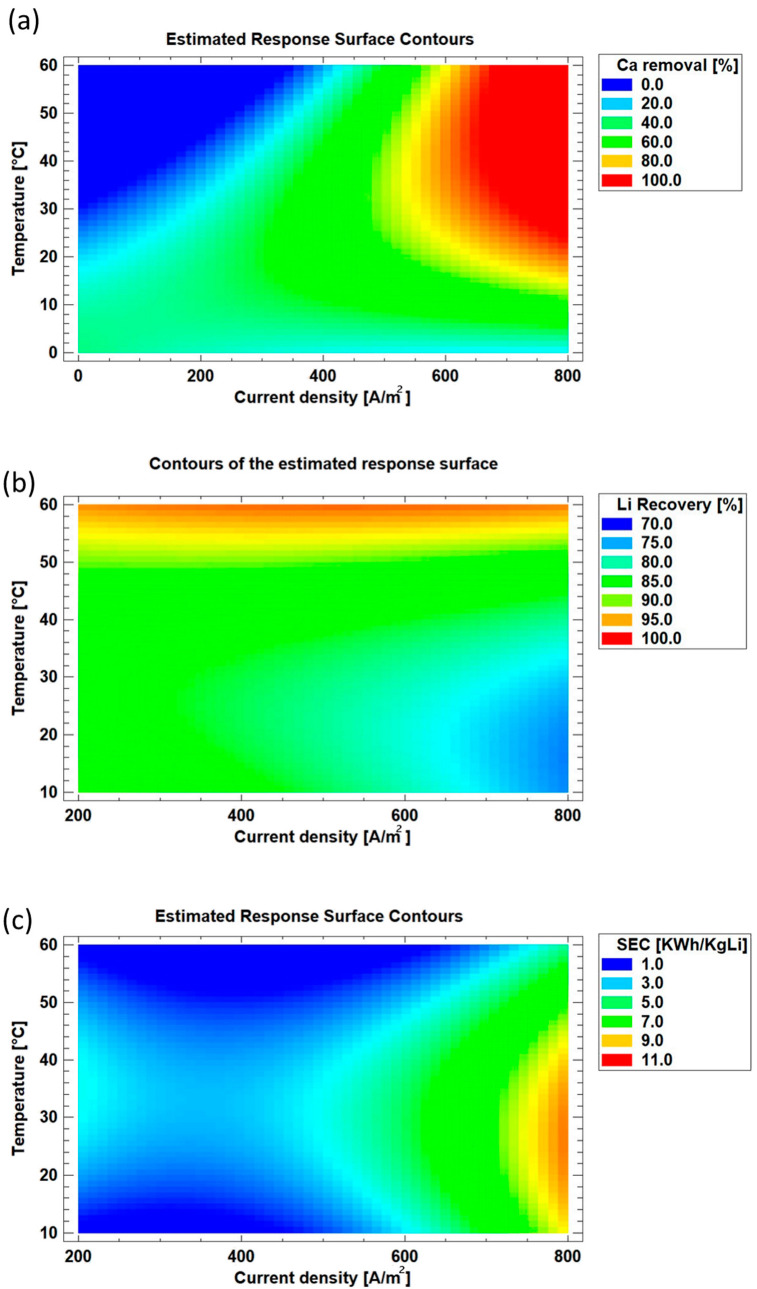
Response surface of (**a**) Ca removal, (**b**) Li recovery, and (**c**) specific electrical energy consumption according to temperature and current density.

The response surface analysis represented in Figure 7a shows that Ca^2+^ removal exceeds 90% in the region where the current density is above 600 A/m^2^ and the temperature ranges from ambient conditions to 60 °C. This result confirms that a sufficiently high current density is required to generate an adequate OH^−^ concentrations in the cathodic compartment to drive precipitation. On the other hand, a higher temperature improves the kinetics of electrochemical reactions, lower the electrolyte viscosity, and improve ionic mobility, further favoring the process [41,42]. Nevertheless, current density and total electric charge applied emerge as the most influential parameter governing Ca^2+^ removal.

Figure 7b illustrates the response surface for lithium recoveries. Recoveries above 90% are observed in a region where the temperatures exceed 50 °C and current density remains below approximately 600 A/m^2^. Beyond this value, further increases in current density begin to reduce this optimal recovery region. This behavior can be explained by the fact that higher current density generates a greater number of particles with high surface charge density as a result of the formation of smaller particles. However, elevated temperatures appear to mitigate this effect by promoting particle growth, reducing total surface area, and, consequently, limiting entrapment and adsorption losses. This trend is consistent with the observed decrease in zeta potential with increasing temperature, which drives the system closer to the isoelectric condition and promotes selective Ca^2+^ precipitation without compromising lithium recovery.

At lower current densities, such as 200 A/m^2^, particle growth is favored, potentially leading to reduced lithium loss. However, such low current density may be insufficient to promote Ca(OH)_2_ precipitation, as observed in the Ca^2+^ removal response (Figure 7a).

Figure 7c shows the response surface for specific electrical energy consumption (SEC). The optimal region, defined by SEC values below 6 kWh per kilogram of Li recovered, is located at current densities below 600 A/m^2^. Operating at temperatures between 20 and 40 °C tends to increase SEC, primarily because at lower temperatures and current densities, Mg^2+^ and Ca^2+^ precipitation remains incomplete. A region of lower specific electrical energy consumption is identified when operating at temperatures above 45 °C.

### 3.6. ANOVA Variance Analysis for Li Recovery and Ca Removal

To quantitatively evaluate the effects of temperature and current density, an ANOVA was performed. A *p*-value < 0.05 was considered statistically significant. In the ANOVA for lithium recovery, the temperature factor showed a statistically significant effect with a *p*-value of 0.0105, below the 0.05 threshold. This indicates that it has a relevant influence on the response variable with 95% confidence. In contrast, current density showed a moderate influence with a *p*-value of 0.0906, which is statistically significant at the 90% level but not at 95%. Neither the quadratic effects of the factors and interaction between temperature and current density were statistically significant as their *p*-values exceeded 0.05, indicating that the interaction temperature–current density does not contribute significantly to the observed variability.

The coefficient of determination (R^2^) was 89.82%, suggesting that the model explains a large portion of the variability in lithium recovery. The adjusted R^2^, which accounts for the number of variables, was 77.09%, reflecting a reasonably good fit. However, the predicted R^2^ reached only 30.24%, revealing limited ability to extrapolate results beyond experimental dataset.

In the case of calcium removal, current density was the only factor with a statistically significant effect (*p*-value = 0.0132), confirming its direct influence on the precipitation. Temperature had no significant influence (*p*-value = 0.3374), nor did the quadratic effects or the interaction between factors, all with *p*-values above 0.05. The fitted model provided an R^2^ of 84.67%, indicating that it explains a large portion of the observed variability. Although the adjusted R^2^ decreased to 65.52%, the model still adequately represents the main effects. However, the predicted R^2^ was low, at 15.07%, suggesting limited predictive capability for results beyond the experimental dataset. The standard error of the estimate was 21.77, and the mean absolute error was 11.28, indicating some variability between observed and estimated values.

In contrast to the above models, the statistical analysis of specific electrical energy consumption (SEC) showed no significant effects on the response variable as none of the factors or their interactions had a *p*-value below 0.05. Although the dataset explained 74.46% of the total variability, its predictive capability was null, with a predicted R^2^ of 0%, and the model fit weakened when accounting for the number of variables, with an adjusted R^2^ of 42.54%. Despite this, the standard errors of the estimate were low, indicating moderate dispersion. The dataset generated is not robust enough to generalize or make reliable predictions regarding specific electrical energy consumption based on the valid experimental data. This may be due to the influence of other unaccounted factors or high variability within the dataset. One explanation is that the applied electric charge differed between samples, and this charge is directly related to SEC. Additionally, unaccounted factors may influence the results, such as the different percentages of Ca removed, which directly affect SEC.

Although the fitted models for Li recovery and Ca removal show high R^2^ values (>75%), their predictive R^2^ is low. The model is not predictive, but it is still useful for qualitative interpretation and identifying key influencing factors.

### 3.7. Challenges and Future Prospects

The zeta potential varied dynamically with the OH^−^ concentration in the brine, which evolved over time based on the applied current density and the concentration of Mg^2+^ and Ca^2+^. Temperature influenced ionic mobility and the nucleation behavior of Mg(OH)_2_ and Ca(OH)_2_, while current density influences the rate of OH^−^ generation at the cathode.

At lower temperatures, slower nucleation favored the formation of larger particles, yet their reduced precipitation rate led to OH^−^ accumulation and relatively high positive zeta potential values. In contrast, higher temperatures accelerated both nucleation and subsequent particle growth while also increasing Mg(OH)_2_ and Ca(OH)_2_ solubility. These combined effects reduced surface charge density and drove the zeta potential toward lower (less positive) values, approaching the isoelectric condition and enabling more efficient Ca^2+^ removal without compromising lithium recovery. Conversely, higher current densities promoted rapid OH^−^ generation and pH elevation, typically resulting in lower zeta potential values due to accelerated precipitation.

While higher zeta potential improves colloidal stability by increasing electrostatic repulsion, it also prevents particle agglomeration, resulting in smaller dispersed particles that hinder sedimentation and complicate solid–liquid separation. Therefore, reaching higher pH values is often necessary to ensure Ca^2+^ removal. However, the associated decrease in zeta potential can lead to particle agglomeration, which may be beneficial for separation processes.

In this study, zeta potential is used primarily as a tool to monitor surface charge evolution and stability trends, rather than a standalone predictor of lithium retention. A complete understanding of adsorption mechanisms would require complementary characterization techniques such as surface adsorption isotherms or XPS analysis.

Interestingly, lower zeta potential values, though indicative of reduced repulsion, can enhance particle aggregation and separation as long as lithium losses remain reduced (test ML6).

Throughout the experiments, the zeta potential remained positive, preventing fouling of the anion exchange membrane. Instead, solid deposition occurred mainly on the electrode surface, underscoring the need for improved hydrodynamic conditions to remove adhered particles and reduce resistive losses.

A high pH is essential to achieve maximum removal of divalent cations as it leads to lower potential and facilitates precipitation. Test ML6 demonstrated high Ca^2+^ removal and low lithium loss by applying excess current; however, this resulted in a low apparent faradaic efficiency. An important opportunity for improvement lies in optimizing the total applied charge, and thus the current density–time combination, to match the stoichiometric OH^−^ requirement for complete Ca^2+^ precipitation. Real-time monitoring of pH or conductivity could allow precise termination of electrolysis at the end of the Ca(OH)_2_ precipitation plateau, preventing excessive OH^−^ generation and improving faradaic efficiency while maintaining high removal rates.

Future studies should explore the development of a continuous, steady-state system with real-time control over operational conditions to minimize lithium losses. Optimizing particle growth and aggregation dynamics could enhance process selectivity and efficiency.

Although the present study was conducted using synthetic brines, the observed trends in zeta potential behavior and selective precipitation mechanisms are likely generalizable to other lithium-rich brines containing Mg^2+^ or Ca^2+^ concentrations. The combined methodology, integrating electrochemical control and zeta potential monitoring, offers a promising framework for lithium brines purification. Future research could extend this approach to systems with varying ionic strengths, membrane configurations, and brine compositions.

## 4. Conclusions

This work demonstrates the feasibility of simultaneous electrochemical removal of Mg^2+^ and Ca^2+^ from lithium-rich brines using a three-compartment membrane cell, achieving high removal efficiency without chemical precipitants. The best result was obtained at 600 A/m^2^ and 50 °C, achieving 99.6% Mg^2+^ removal, 99.5% Ca^2+^ removal, and over 90% Li^+^ recovery, with a specific electrical energy consumption of 2.58 kWh/kg Li. Although the best overall performance was achieved in ML6, the results indicate that energy consumption could be further reduced by optimizing the applied charge to the stoichiometric requirement for complete precipitation. These results highlight the potential of the method but remain specific to the operational window explored. Further validation under variable brine compositions and flow regimes is needed.

Zeta potential measurements indicated that Mg(OH)_2_ and Ca(OH)_2_ particles exhibited a positive surface charge (4–20 mV), falling below the commonly accepted threshold (±30 mV) for stable suspensions. Zeta potentials with an absolute value lower than 30 mV generally indicate weak interparticle repulsion and a tendency for particle agglomeration, which in our case could trap brine within solid aggregates. Such entrapment could account for lithium losses due to physical retention.

Calcium removal was found to be more sensitive than magnesium to variations in current density and temperature. Higher temperatures decrease the pH required for precipitation, while higher current densities accelerated OH^−^ generation, enhancing precipitation kinetics. Lithium recovery of 90% and a high degree of calcium removal were achieved under conditions that favored the formation of weakly charged particles.

The process yielded purified brines with enhanced Li/Mg and Li/Ca ratios while preserving monovalent cation concentrations. Additionally, the migration of Cl^−^ across the anion exchange membrane enabled in situ generation of HCl, reaching concentrations up to 3.8 M.

Although this study was conducted with synthetic LiCl brines containing controlled impurity levels, the operational framework developed here is expected to be applicable to real-world brines. However, natural brines often present additional complexity, including other dissolved species (e.g., borates, sulfates, and transition metals), variable ionic strength, organic matter, and seasonal or process-related fluctuations in composition. These factors can influence precipitation kinetics, membrane transport, and surface charge evolution, potentially shifting the optimal operating window identified in this study.

Future work should focus on scaling the system to continuous steady-state operation using real brine matrices. A deeper investigation into surface interactions and lithium adsorption mechanisms is required to further minimize lithium losses and improve scalability.

## Figures and Tables

**Table 1:** Chemical compositions of initial LiCl solution and concentration ratios of lithium over other cations.

[ppm]	Li	Ca	Mg	Na	K	Li/Mg	Li/Ca	Li/Na	Li/K
Li solution	11,600	1260	440	492	982	26.36	9.21	23.58	11.81

**Table 2:** RSM experimental design with star configuration.

Test ID	Current Density (A/m^2^)	Temperature (°C)
ML1	400	35
ML2	400	35
ML3	200	20
ML4	600	20
ML5	200	50
ML6	600	50
ML7	117.2	35
ML8	682.8	35
ML9	400	13.8
ML10	400	56.2

**Table 3:** Ratios of Li and other ions obtained in the final brine.

	Li [%]	Na [%]	K [%]	Li/Mg	Li/Ca	Li/Na	Li/K	pH
Initial Li solution	1.16	0.049	0.098	26.36	9.21	23.58	11.81	1.2
ML1	1.17	0.050	0.092	5850	34.82	23.64	12.72	11.0
ML2	1.18	0.050	0.105	5900	13.30	23.98	11.20	11.0
ML3	1.13	0.049	0.088	5650	13.97	23.25	12.84	11.5
ML4	1.12	0.048	0.098	5600	30.94	23.24	11.39	12.0
ML5	1.39	0.065	0.116	6950	11.32	21.41	11.94	10.4
ML6	1.57	0.073	0.132	7850	2617	21.42	11.84	12.0
ML7	1.16	0.049	0.098	5800	9.15	23.63	11.81	10.5
ML8	1.21	0.046	0.096	6050	103.4	24.44	12.57	11.7
ML9	1.17	0.048	0.094	5850	18.17	24.27	12.43	12.0
ML10	1.27	0.058	0.105	6350	10.61	22.09	12.05	10.0

## Data Availability

The original contributions presented in this study are included in the article. Further inquiries can be directed to the corresponding author.
